# The Event Detection System in the NEXT-White Detector

**DOI:** 10.3390/s21020673

**Published:** 2021-01-19

**Authors:** Raúl Esteve Bosch, José F. Toledo Alarcón, Vicente Herrero Bosch, Ander Simón Estévez, Francesc Monrabal Capilla, Vicente Álvarez Puerta, Javier Rodríguez Samaniego, Marc Querol Segura, Francisco Ballester Merelo

**Affiliations:** 1 Instituto de Instrumentación para Imagen Molecular (I3M), Centro Mixto CSIC—Universitat Politècnica de València, Camino de Vera s/n, 46022 Valencia, Spain; jtoledo@eln.upv.es (J.F.T.A.); viherbos@eln.upv.es (V.H.B.); vialpue@upv.es (V.Á.P.); jarodsa@upv.es (J.R.S.); fballest@eln.upv.es (F.B.M.); 2 Nuclear Engineering Unit, Faculty of Engineering Sciences, Ben-Gurion University of the Negev, P.O. Box 653, Beer-Sheva 8410501, Israel; ander@post.bgu.ac.il; 3 Donostia International Physics Center (DIPC), Paseo Manuel Lardizabal 4, 20018 Donostia-San Sebastian, Spain; francesc.monrabal@dipc.org; 4 IKERBASQUE, Basque Foundation for Science, 48013 Bilbao, Spain; 5 Instituto de Física Corpuscular (IFIC), CSIC & Universitat de València, Calle Catedrático José Beltrán 2, 46980 Paterna, Spain; marc.querol@ific.uv.es

**Keywords:** xenon TPC, trigger concepts, data acquisition circuits, FPGA

## Abstract

This article describes the event detection system of the NEXT-White detector, a 5 kg high pressure xenon TPC with electroluminescent amplification, located in the Laboratorio Subterráneo de Canfranc (LSC), Spain. The detector is based on a plane of photomultipliers (PMTs) for energy measurements and a silicon photomultiplier (SiPM) tracking plane for offline topological event filtering. The event detection system, based on the SRS-ATCA data acquisition system developed in the framework of the CERN RD51 collaboration, has been designed to detect multiple events based on online PMT signal energy measurements and a coincidence-detection algorithm. Implemented on FPGA, the system has been successfully running and evolving during NEXT-White operation. The event detection system brings some relevant and new functionalities in the field. A distributed double event processor has been implemented to detect simultaneously two different types of events thus allowing simultaneous calibration and physics runs. This special feature provides constant monitoring of the detector conditions, being especially relevant to the lifetime and geometrical map computations which are needed to correct high-energy physics events. Other features, like primary scintillation event rejection, or a double buffer associated with the type of event being searched, help reduce the unnecessary data throughput thus minimizing dead time and improving trigger efficiency.

## 1. Introduction

NEXT-White [1] is the phase one of NEXT-100 [2], an experiment to measure the neutrino double beta decay. The main objectives of NEXT-White are to demonstrate the High-Pressure Xenon Gas (HPGXe) TPC technology and to measure and validate the background model for the next generation of NEXT (Neutrino Experiment with a Xenon TPC) detectors. NEXT is a staged experimental program aiming at the detection of ββ0ν in 136Xe, using successive generations of high-pressure gaseous xenon electroluminescent Time Projection Chambers (HPXe EL-TPC). While other xenon experiments (EXO [3], KamLAND-Zen [4]) have an approach of large exposures at the cost of losing energy resolution (and therefore background rejection potential), the HPGXe TPC technology has demonstrated an excellent energy resolution (1% FWHM at the Q value of the double beta decay [5]) and an impressive topological rejection factor thanks to the relatively low density of the xenon gas and to the tracking system with SiPMs [6]. These two tools provide a very low background index in the region of interest near Q_ββ_, a fundamental parameter for next generation detectors.

NEXT-White uses a 5 kg high-pressure gaseous xenon TPC with electroluminescence (EL) charge amplification and optical readout. The detector, a prototype of NEXT-100, is based on a PMT plane for energy measurements and a SiPM tracking plane for topological event filtering. The experiment has been operating since the end of 2016 at the Laboratorio Subterráneo de Canfranc (LSC) in Spain and will end its data-taking and be replaced by NEXT-100, during 2021. The NEXT-White detector, being a demonstrator of the 100 kg scale NEXT-100 detector, is needed to prove all the technical solutions that will be implemented in the later detector. Specially, the background budget of the different materials and components was strictly controlled to minimize it.

**Figure 1:**
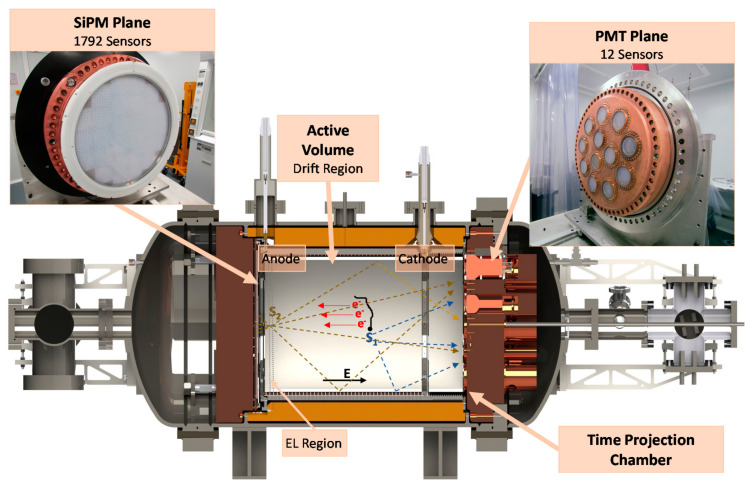
Schematic view of the NEXT-White Detector. Top right: Picture of the PMT sensors plane. Top left: Picture of the SiPM sensors plane. In the active volume of the TPC: Drawing with the principle of operation of the detector.

NEXT-White is an optical TPC. Ionizing radiation excites and ionizes the gas producing primary scintillation (S_1_) and ionized electrons. The presence of a moderate electric field in the active volume prevents their recombination, drifting them towards the amplification region near the anode (EL region). In the anode, a more intense electric field accelerates the electrons producing a secondary scintillation (S_2_) as a result of an amplification process. The light is detected with two sensor planes in opposite sides of the detector vessel (see Figure 1). The PMT plane measures event energy by integrating the amplified S_2_ signal with 12 PMTs (Hamamatsu R11410-10) with a total area coverage of 31%. This plane is also responsible for the detection of the primary scintillation light that provides the event t0 and allows to position the event in the axial axis of the detector [7,8]. The SiPM Plane, instrumented with 1792 SiPMs (SensL series-C) in an array at a pitch of 10 mm, detects the light produced in the EL region allowing topological reconstruction that helps to discriminate double beta decay events from the background, being a unique characteristic of pressure gas detectors.

The Data Acquisition System (DAQ) in NEXT-White is an enhancement of the system developed for the NEXT-DEMO detector [9], the first prototype of NEXT-100. A double data circular buffer has also been implemented to reduce dead time. DAQ also implements data compression to reduce data throughput, which also has a remarkable impact on decreasing overall system dead time.

Event detection systems for argon or xenon TPCs such as those from LUX, LZ, DARKSIDE-50 and PANDA [10,11,12,13], as well as NEXT-White TPC, are based on fast detection events (S_1_, S_2_, or both) either per PMT, or using a signal sum of several PMTs. The detection operation is carried out by means of temporal, amplitude or energy parameters. Moreover, it is common to have a second level of detection that requires the coincidence of hits in a time window. In addition to these, the event detection system described in this article brings some relevant and new functionalities. First, it adds more complex event selection flexibility since the S_2_ expected signals generated by MeV electrons in NEXT-White are very diverse due to the large variety of possible topologies of the events. Second, the system allows to carry out calibrations while taking physics data, ensuring a dynamic calibration. And third, the double circular buffer can be prioritized for a type of event thus reducing dead time for this selected event, generally, physics data versus calibration data.

## 2. Event Detection in NEXT

NEXT-White can provide a measurement of the two-neutrino double beta decay mode. Furthermore, to fully validate the detection technique, the NEXT collaboration must perform several studies in three different areas. First, the comparison of the background model extracted from in situ data-taking with the expected contribution to the radioactive budget of the different components. Second, to determine the energy resolution that must be in the order of 1% FWHM for energy depositions near the Q-value of the decay (Q_ββ_), that is 2.458 MeV. And third, the background rejection efficiency of the topological signature characteristic of this type of TPC.

Detector calibration and definition of the background model requires the detection of events of different characteristics. Alpha, electron, gamma, x-ray events associated with diverse sources (^222^Rn, ^137^Cs, ^232^Th, ^22^Na, and ^83m^Kr) and muons, together with distinct field settings, have been studied to understand the detector in a wide range of energies, as well as to measure its energy resolution [5,14,15,16]. Equalization of the PMT gain is also foreseen in dedicated runs by means of regular LEDs that generate pulses periodically.

The event detection is performed by reading the signal of the PMT sensors. As commented in Section 1, one of the strengths of the NEXT technology is the capability to reconstruct the topology of the electrons in the gas. In a TPC, different topologies imply different temporal distributions of the charge reaching the amplification region and thus different amplitude and time duration pulses in the PMT sensors.

**Figure 2:**
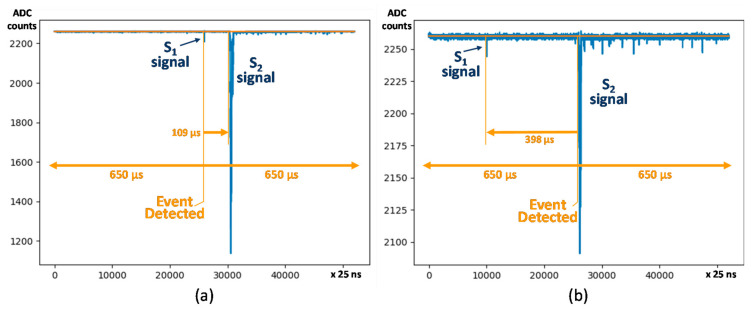
NEXT-White most common signal searches: (**a**) Online S_1_ signal search, with offline S_2_ signal search; (**b**) Online S_2_ signal search with offline S_1_ signal search. In both cases, a data acquisition window of 1300 µs and pre-trigger of 650 µs is applied.

In order to identify diverse types of events, different event detection approaches have been explored. Figure 2 shows the typical type of searches applied in the experiment. In the most common approach, DAQ must identify online S_2_ signals in a set of PMT sensors synchronously. The S_2_ signal is centered in the middle of the data acquisition time window, while the pre-trigger data shows the S_1_ signal, indicating the time drifted by the electrons that have a linear relation with their initial position in the detector, thus allowing for a 3D reconstruction of the event. In this approach, a peak-finding offline algorithm searches for the S_1_ that can be as small as one photoelectron per PMT. In other approach, the same identification procedure is used to identify synchronously S_1_ signals online in a set of PMT sensors, searching also for the corresponding S_2_ offline. Both approaches are valid for detector calibration and physics data.

External trigger and a set of LEDs arranged in the chamber planes are also needed for test purposes and gain equalization of the different types of sensors.

## 3. NEXT-White Data Acquisition Hardware Architecture

### 3.1. Hardware Architecture

NEXT-White DAQ System, described in [17], is based on the SRS-ATCA (Scalable Readout System ported to the Advanced Telecommunications Computing Architecture standard) jointly developed by the NEXT Collaboration, CERN-PH and IFIN-HH Bucarest in the framework of the CERN RD51 collaboration [18,19]. Based on FPGA, it provides a customizable interface between a wide range of front-end and DAQ. Originally developed for micropattern gaseous detectors (MPGDs), it has also been used with other sensors such as PMTs and SiPMs. This flexibility is achieved via generic plug-in cards to a common module for all applications, the SRS DAQ module. The SRS default online system for RD51 users is DATE (ALICE Data Acquisition and Test Environment) [20], though other software options are possible, like RCDAQ [21,22].

**Figure 3:**
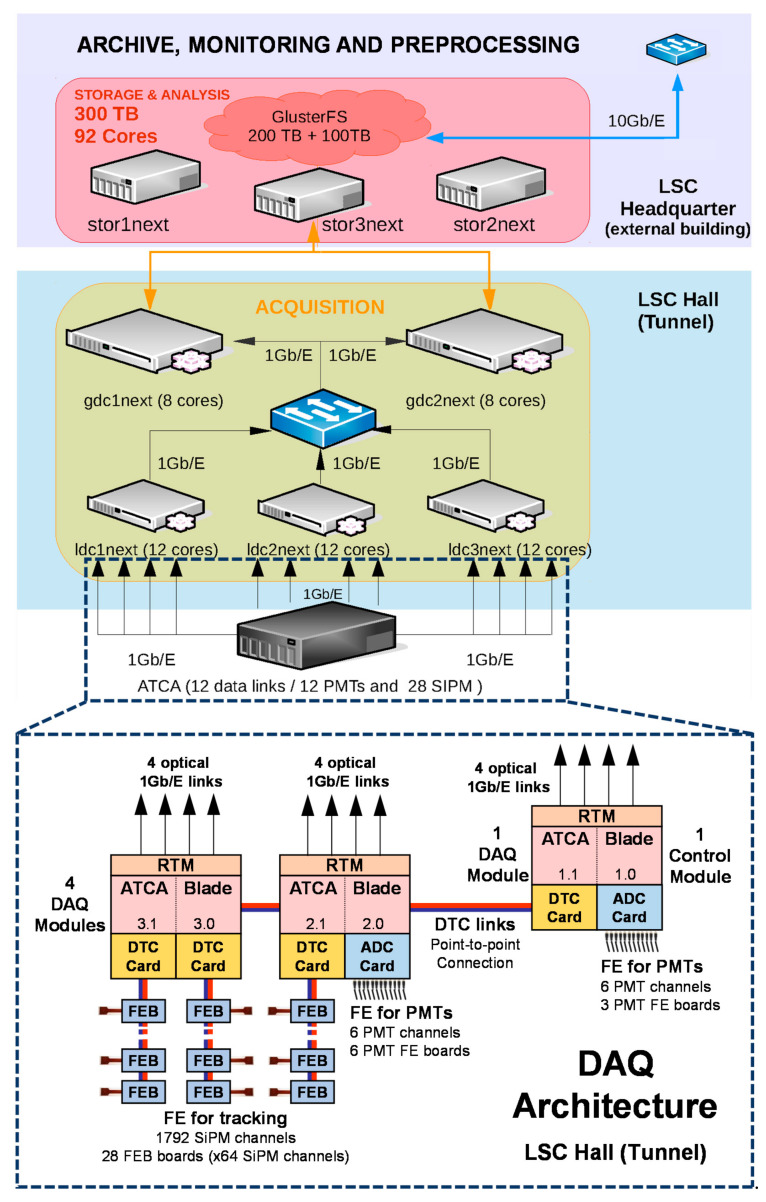
NEXT-White Data Acquisition Hardware Architecture.

The SRS DAQ module (referred to as “ATCA blade” in Figure 3) provides real-time digital processing through two Xilinx Virtex-6 FPGAs (XC6VLX240T-1ff1156). Each FPGA is connected to a DDR3 SO-DIMM memory module that can be used as a data memory buffer. Two on-board custom mezzanine connectors provide I/O flexibility for a wide range of front ends. We use two different mezzanines: the EAD-M1 unit (ADC Card), with 24 ADC channels (12 bit, up to 60 MSa/s) and the DTC card, with 12 DTC links [23] on HDMI connectors to interface digital front ends at a maximum speed of 200 Mb/s. DTC stands for Data Trigger and Control and was specified in the framework of RD51′s SRS as a 4-pair differential interface between digital front-ends and the SRS DAQ module. It can be used for configuration and data transfer, and trigger purposes. The SRS DAQ module also includes connection to a Rear Transition Module (RTM) for multiple GbE, 10 GbE and other I/O connectivity, like two HDMI connectors for DTC connection and external input/output NIM connection.

In the case of NEXT-White, on the one side, the SiPM plane is readout through the SiPM Front-End Board (FEB) [24] and a DTC link to the SRS DAQ module. FEBs provide the analogue front-end electronics for 64 SiPM sensors and digital processing through a Xilinx Virtex-6 FPGA (XC6VLX130T-1ff784). On the other side, the PMT plane analogue front-end is interfaced directly through the EAD-M1 unit, that is used to digitize the analogue signals of the PMT sensors. In both cases, sensor data are sent individually to the DAQ PCs for offline processing.

### 3.2. System Architecture

As shown in Figure 3, three SRS DAQ modules (in the figure ATCA blades) are needed to read out the data from PMT and SiPM sensors, as well as to perform the event detection and system control. In NEXT, each SRS DAQ module constitutes two independent modules, five of them are used mainly for data acquisition purposes (DAQ Modules) and one for system control purposes (Control Module). DAQ modules devoted to readout PMT sensors can readout up to 12 PMTs each, while DAQ modules devoted to read SiPM sensor can readout data from 12 FEB boards each. Since each FEB board digitizes, formats and sends data form 64 SiPM sensors, 28 boards are enough to readout the total amount of sensors needed, 1792 SiPM sensors.

For system control duties, the Control Module is connected with the other modules through four LVDS-pair point-to-point connections (DTC links) devoted to clock distribution, synchronization, system configuration and event command handling. DTC links are also used to connect DAQ Modules to FEBs through DTC cards.

The readout works in push mode. DAQ Modules read out, time stamp and store data coming from the front-end in a reconfigurable-length circular buffer, whose maximum size corresponds to approximately six times the maximum detector drift time (up to 3200 µs). The circular buffer is indeed a double circular buffer implemented on DDR3 memory.

Data are sent from the DAQ Modules to the DAQ PCs using optical 1 Gb/E links. The online system, DATE, defines a hierarchy in the DAQ computers in which Local Data Concentrator (LDC) PCs receive data that is merged in sub-events and sent to a set of Global Data Concentrator (GDC) PCs. GDCs send complete events to the final storage.

DAQ Modules can be configured to limit data throughput. This feature prevents data loss and also limits the number of LDC and GDC PCs needed. In NEXT-White three LDCs and two GDCs suffice to handle the event data load of 120 MB/s initially set. Data acquired near the detector is sent outside the LSC Hall to an external building where data are analyzed and processed in a set of servers before being sent to final storage on tape.

A JAVA GUI implemented in the System Configuration PC is used to configure and control the complete system through the Control Module. All the modules also send slow control information that is processed by the GUI and sent to the detector Slow Control System.

## 4. Event Detection System

### 4.1. Event Detection Architecture

**Figure 4:**
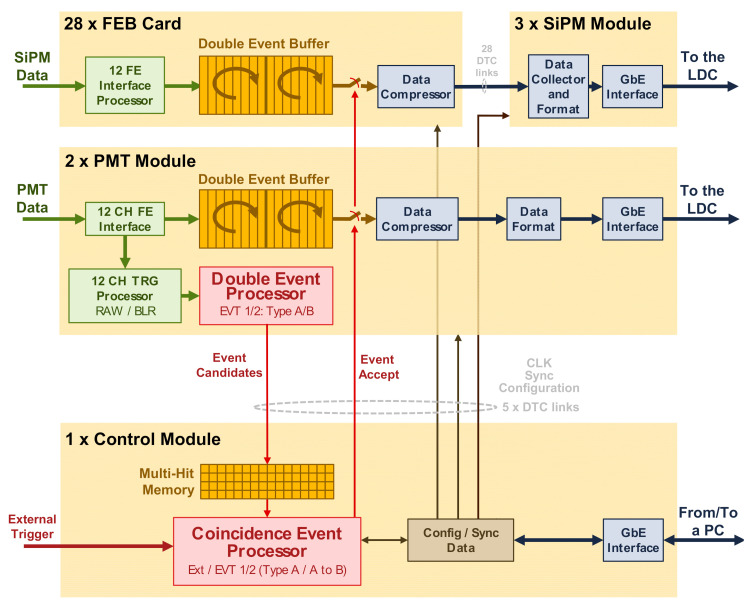
NEXT-DEMO trigger scheme.

In NEXT-White, event detection is based on two processing levels, distributed among different modules, as shown in Figure 4. In the first level, from the early energy measured in the PMT sensor, the Double Event Processor (DEP) generates event candidates, which are sent to the second level, located in the Control Module. In this module, event candidates are stored in the Multi-Hit Memory to be later processed by the Coincidence Event Processor (CEP). This processor generates an Event Accept signal that produces data upload from each DAQ Module buffer to the online system for later offline analysis.

Both DEP and CEP have a two-processor based parallel architecture. Each pair of processors can be configured with a set of parameters to search for a different type of event marked as EVT1 or EVT2 type. As mentioned in Section 2, the most obvious application of these double event detection feature is to permit calibrations to be carried out while taking physics data, ensuring high-quality and properly calibrated physics data. In this context, EVT1 type is generally set to detect calibration events, while EVT2 type is devoted to physics events.

As described in Section 3.2, DAQ data storage is partitioned into two circular buffers. A central memory state machine manages the assignment and disposal of the buffers and controls the memory readout. Sampled data are stored in the first available buffer. Moreover, the use of the double circular buffer can be associated to the type of event, allowing two modes of operation:

- Buffer Mode 1: Every type of event detected, EVT1 or EVT2 type, uses the double buffer indistinctly. No priority is given no any event type.
- Buffer Mode 2: EVT1 type events only use one buffer. If at least one buffer is full, the free remaining buffer is always reserved for the accommodation of EVT2 type events. That means that priority is given to EVT2 type events. In this mode of operation, EVT2 type is devoted to detect physics data since it guarantees the minimum dead time for this type of event.

### 4.2. External Trigger

The DAQ has a third mode of operation for external trigger and test purposes called Test Mode. When this mode is set, event type configuration is not relevant and event processors are set off. In this mode, when an external NIM pulse is detected, the Event Accept is generated automatically. The trigger can be also be internally generated and configured in frequency, so the external NIM trigger source is not needed.

In NEXT, the external trigger is used to calibrate the detector sensors. The aim of the calibration is to equalize precisely the gain of the sensors. NEXT-White sensors are calibrated using LEDs located in the PMT and the SiPM planes. In both type of sensor calibration, the light of the LEDs is adjusted to have a response in the sensors on the order of the single photo-electron.

In the case of the SiPM sensors, the LED, placed in the opposite plane, is connected to a pulse generator outside the detector which generates synchronously the NIM external trigger signal. This configuration allows acquiring data only in a window where the LED is active. PMT sensors are calibrated with a similar procedure but, in this case, the LED is placed in the SiPM plane and is controlled through the FEBs. No external NIM input is needed since the Event Accept generation is internally synchronized with the signal needed for the LEDs.

In both cases, since the signal under study is related to the single photo-electron signal, the buffer needed can be of the order of hundred microseconds. On the one side, this configuration strongly accelerates the acquisition rate as the data throughput is highly reduced, compared to the usual data-taking. On the other side, it ensures that the signal induced by the LED is in a fixed time position defined by the acquisition system facilitating the data processing performed offline.

### 4.3. The Double Event Processor

#### 4.3.1. Event Detection

**Figure 5:**
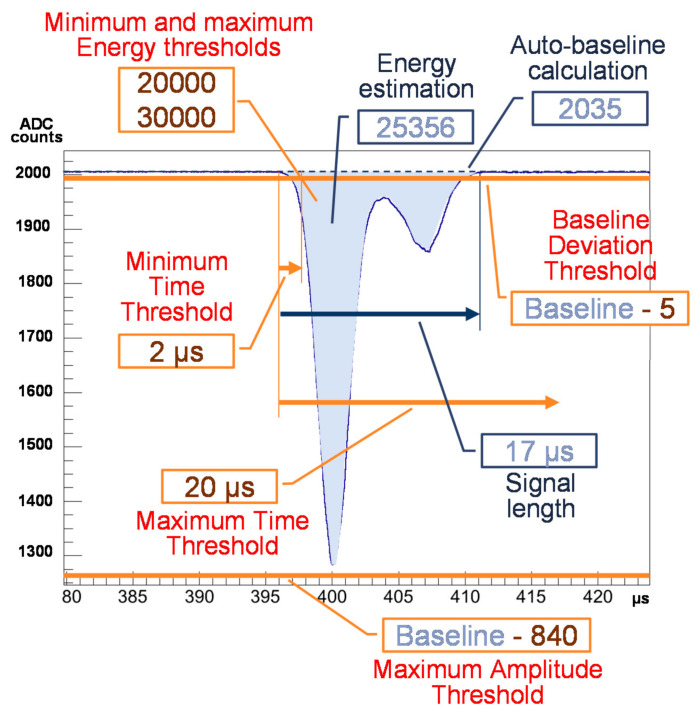
Example of signal candidate generation. In red, the complete set of configuration parameters to generate an event candidate from a PMT signal. In blue, data estimated by the event processor over the reconstructed signal by the BLR algorithm.

As shown in Figure 5, each of the Double Event Detection processors generates event candidates per selected PMT channel by means of configurable thresholds on amplitude (baseline deviation and maximum amplitude relative to the self-calculated baseline), on energy (maximum and minimum energy), and time (minimum and maximum length of the event). Different configuration can be applied per PMT channel. Start and end of the signal under study is defined by the baseline deviation threshold.

Event timing is defined by the 40 MHz DAQ master clock generated in the Control Module and it is distributed among DAQ Modules. This frequency matches with that of the PMT signal sampling rate.

Due to capacitive coupling of the PMTs signal, the baseline needs to be restored by a baseline restoration (BLR) algorithm. The effect of these DC rejecting capacitors on the obtained analogue signals is similar to that of a high pass filter with a very low cutoff frequency. Thus, the algorithm being implemented on the Event Detection block inside the FPGA, aims at applying the inverse function of that high pass filter to the digitally acquired signal from the PMT front end, described in [25]. A careful selection of the numerical precision required for the digital implementation and a proper individual calibration of every PMT channel allow a reconstruction of the original PMT output signal with a neglectable error.

A configurable moving average filter applied to the output of the baseline restoration block calculates its baseline value. Energy estimation is computed from the accumulation of the difference between the signal and the calculated baseline whenever the signal is beyond the baseline deviation threshold (noise is below 1 ADC count). The signal length is also estimated. Comparing the estimated values with the aforementioned thresholds in time, energy and amplitude, the event processor establishes whether the current signal is a valid event candidate or not.

Table 1 shows the parameters applied to detect different calibration sources, muons and beta decay physics. This set of parameters allows physic studies for low energy in the range of tens of keV, while for high energy, from hundreds of keV to over 2.65 MeV.

**Figure 6:**
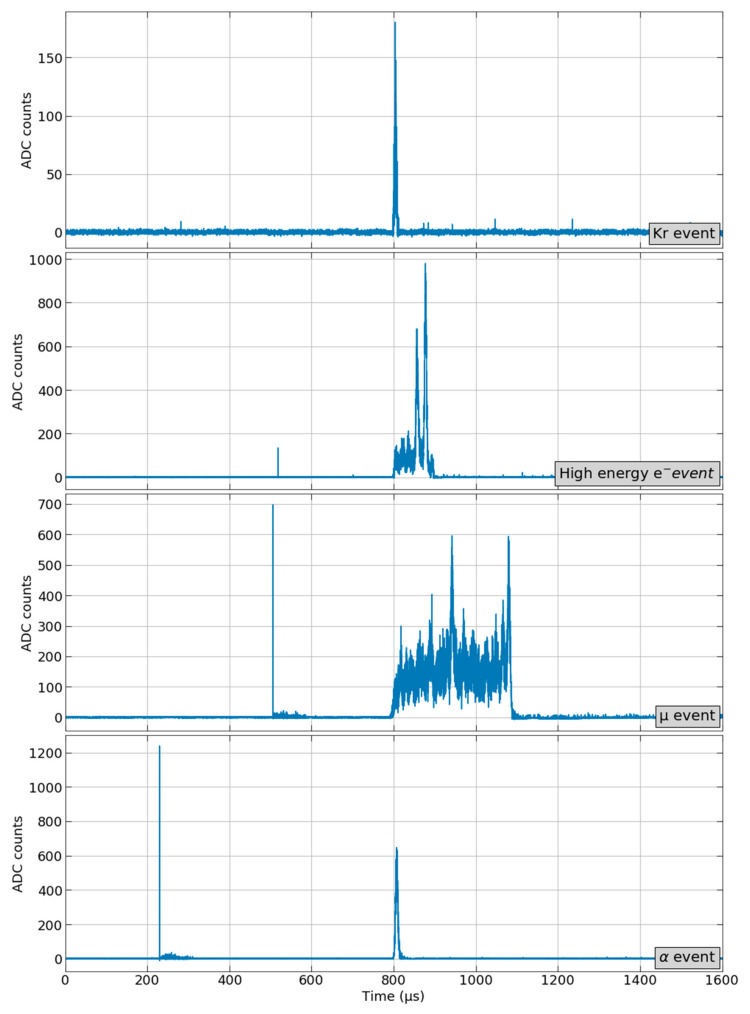
Set of events with a different range of energies from RUN 8250. General configuration: Circular Buffer size of 1600 µs and pre-trigger of 800 µs. EVT1 type set for low energy: Maximum amplitude of 1000 ADC counts, minimum and maximum amplitude thresholds of 5000 and 50,000 sum of ADC counts, and minimum and maximum time thresholds of 2 and 40 µs. EVT2 type set for high energy: Maximum amplitude of 4095 ADC counts (maximum possible value), minimum and maximum amplitude thresholds of 50,000 and 16,777,215 (maximum possible value) sum of ADC counts, and minimum and maximum time thresholds of 2 and 600 µs.

Figure 6 shows a set of different event candidates for a RUN with a different range of energies. In this case, EVT1 and EVT2 types are set for low and high energy event detection respectively. The Kr event corresponds to an EVT1 type event, and the high energy electron, the muon and the alpha events correspond to EVT2 type events.

#### 4.3.2. S_1_ Event Rejection System

As mentioned in Section 2, one of the aims of the NEXT DAQ is to be able to identify S_1_ signals online in a set of PMT sensor synchronously and, in this case, to search for the corresponding S_2_ offline afterwards. This is challenging due to the low amplitude of the S_1_ signal, especially for low energy events.

**Figure 7:**
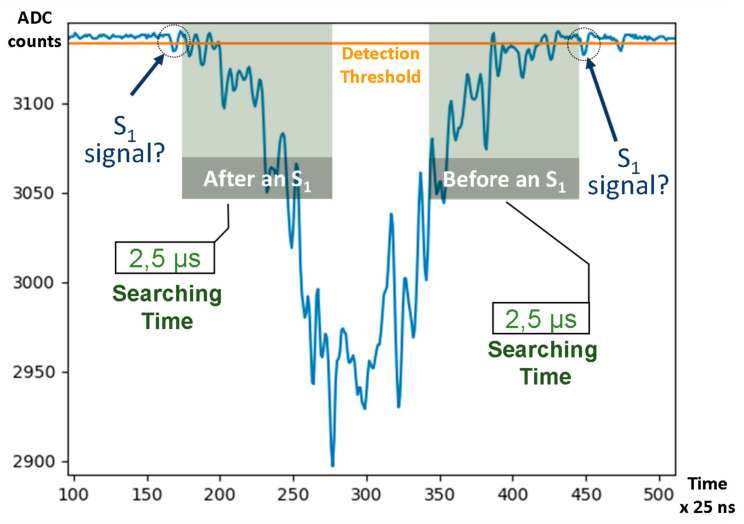
Run 7979 ^83m^Kr energy deposition signal (S_2_) in PMT0 with possible false S_1_ signals after and before the S_2_ signal that could be set as possible event candidates.

When searching for S_1_ signals, due to the topology of the signal generated by some radioactive sources used for calibration, a high number of non S_1_ hits are detected as signal candidates. Some of the runs studied have up to 25% of “false” S_1_ signals. This happens mostly at the beginning or at the end of S_2_ signals, as shown in Figure 7. S_1_ signal hits can be tagged as false due to their topology or other effects that produce small signals too close to an S_2_ signal to be considered a true S_1_.

The Double Event Processor has an additional module to reject the above mentioned false S_1_ signals. When this system is activated, if an S_1_ signal is a valid event candidate, it automatically searches for possible S_2_ signals in a time window around the time of the detected event candidate. In this special case, if the S_2_ signal is detected, the previously selected event candidate is rejected. The rejection time window is configurable in length, as well as the time around the detected event candidate to start the search.

### 4.4. The Coincidence Event Processor

#### 4.4.1. Event Accept Generation

The global Coincidence Event Processor (CEP) centralizes the searching of events in the Control Module. This extra discrimination level is necessary to reject spurious events in individual channel. Two more levels of discrimination are available. First, it is possible to configure searches over a minimum number of event candidates per type in a time window (Coincidence Window). Searches can involve from one to all of the PMTs hits, and the Coincidence Window can be extended up to 1.6 µs. And second, two type of search can be configured since the processor can search for only one signal, or for two following signals in a defined time. These two different types of search give rise to two modes of operation:

1. Single Searching Mode.

This mode is oriented to the search of S_1_ or S_2_ signals. A set of S_1_ or S_2_ signals in a coincidence window generate an Event Accept. Signals must be of the same type (EVT1 or EVT2), but both can be searched in parallel. Priority is given to EVT2 events if both are detected simultaneously.

2. Double Searching Mode.

This mode is oriented to the search of events, that is, an S_1_ signal followed by the corresponding S_2_ signal. A double search implies that the system must be able to define which event must be detected first and which one after. In the case of this searching mode, in the Double Event Processor, event candidates can also be defined as Type A or B, having to be detected in this order. Generally, Type A represents an S_1_-like signal and Type B is an S_2_-like signal. The Event Accept is defined as a combination of the two-consecutive type of signal detected. Strictly, the Event Accept is generated when a set of Type A detected signals is followed, inside a window time, by a set of Type B detected signals, which can be different. In other case, the system looks for another set of type A signals, starting the Event Accept searching process again.

In the case of Single Searching Mode, only Type A signals defined are used by the CEP to generate an Event Accept. Type B signal configuration is ignored by the processor.

**Figure 8:**
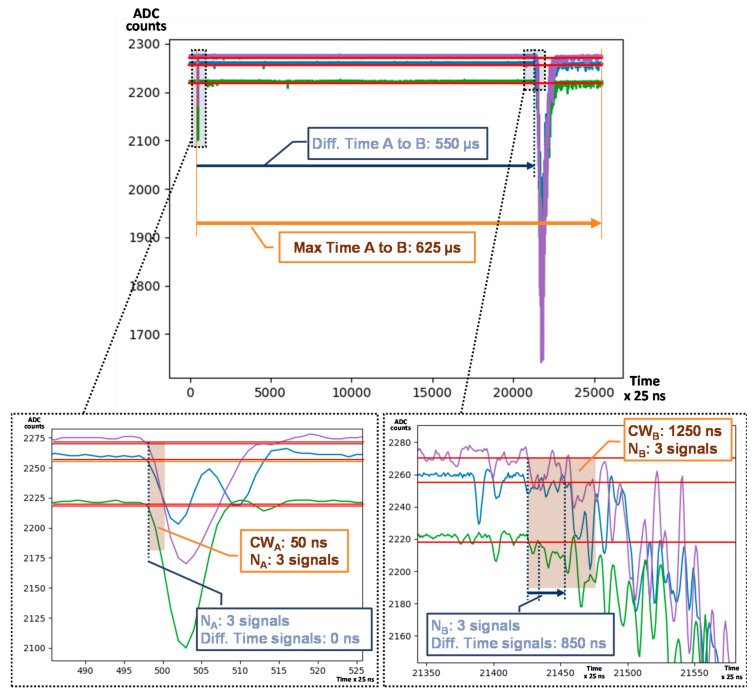
Event Accept example in Double Searching Mode: Run 4405 electron like Type A signal followed by a Type B signal for PMT0, PMT1 and PMT2. Double Search configuration set: 625 µs Maximum Time Event A to B. Type A signal configuration set: 50 ns Coincidence Window (CW_A_) and 3 minimum number of PMT hits (N_A_). Type B signal configuration set: 1250 ns Coincidence Window (CW_B_) and 3 minimum number of PMT hits (N_B_).

Figure 8 shows an example of double search that generates an Event Accept. In blue, number of events and time measured; in brown, configuration required for an Event Accept. For each type of signal, if more than one PMT is involved, the starting time is set by the first detected hit among them. Once a set of signals satisfies Type A conditions (at least 3 signals in the same time bin in the example), the processor looks for Type B signal candidates. As seen in the figure, in less than 625 µs (maximum difference allowed between Type A and B signals), at least 3 Type B signals are detected in 850 ns, satisfying Type B proposed conditions and, therefore generating an Event Accept.

Since PMT sensors can be readout by different DAQ Modules, event timing is defined by the 40 MHz DAQ master clock generated in the Control Module as previously stated in Section 4.3.1. This clock is distributed through the DTC links and synchronized at the level of a clock period among modules.

Statistics per event type of the Event Accept finally processed and lost are continuously sent to the offline system for later analysis of the background rate and half-live of the two neutrino double beta decays over the time for a run.

Events lost occurs when an Event Accept is rejected in some conditions. Independently of the searching mode, when an Event Accept is generated, processing continues and no other events are accepted until the buffer is complete and ready to be downloaded. In this special case, new Event Accept are rejected but do not contribute to the event lost statistics. However, when buffers are full, Event Accepts are also rejected and counted as lost events. In the case of the buffer full condition, the system will reject Event Accepts depending on the Buffer Mode selected and event type detected, as described in Section 4.1.

#### 4.4.2. Multi-Hit Memory Concept

DAQ Modules are continuously sending event candidates to the Control Module. Event candidate related data, time-stamp and channel number, are stored in a Multi-Hit Memory that is implemented in a true dual port RAM. Each of the available Multi-Hit Memories (one per type of event) has the size, or an exact divisor, of the programmed Circular Buffer, allowing multiple configurations in size of the Circular Buffer. The Multi-Hit Memory stores, per cell, the number of possible hits, type A or B for double search, and channel hit, with the minimum number of bits to reduce the data memory needed.

Each memory data cell of a Multi-Hit Memory points to a timer related value. This timer is the Fine Timer (FT). The FT is connected to the 40 MHz DAQ master clock. The FT synchronizes event time-stamps and event accept timing among modules.

**Figure 9:**
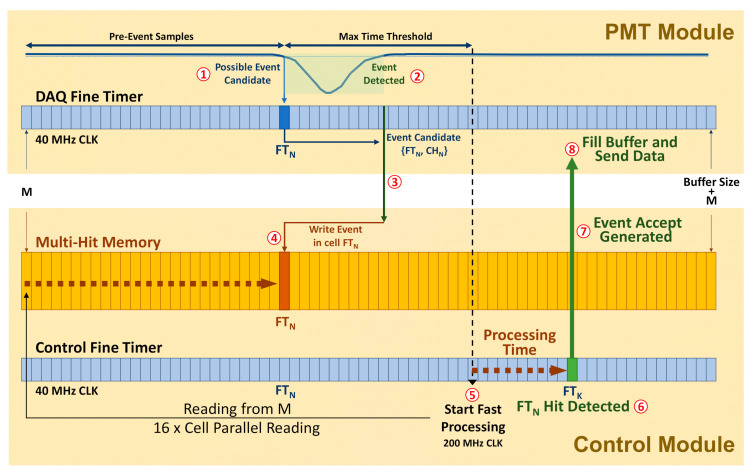
System event detection and Multi-Hit Memory scheme and functionality example.

As shown in Figure 9, when a signal is detected, the system performs a set of steps to generate an Event Accept:

1. When a signal is below the threshold set (Baseline Deviation Threshold), the event time-stamp and channel are stored: FT_N_ and CH_N_ respectively.
2. When the signal is above the Baseline Deviation Threshold, estimated values are checked. If the signal satisfies the configuration set, the event is considered as detected, if not, event candidate data stored are discarded.
3. The event candidate data are sent to the Control Module. Event data transmission and memory writing take around 2 µs.
4. Event candidate’s data received in the Control Module are stored in the Multi-Hit Memory in the correspondent position to FT_N_ in the memory. As mentioned above, DAQ and Control Timers are synchronized by the DAQ system master clock.
5. In order to guarantee that the complete signal under study is stored, an amount of time must elapse prior to starting processing the hits. The time elapsed must be at least the time corresponding to the configured buffer pre-samples and maximum time threshold.
6. It is then when processing starts from the beginning of the memory. To speed up processing, the Coincident Event Processor runs at 200 MHz. Moreover, groups of 16 memory cells are read out and stored in a shift register performing a flash detection in one clock cycle. Moreover, only one clock cycle is needed to read and clear the cells. In the case a hit is found, it takes 16 cycles to process the shift register. Processing implies searching for other hits and getting the total number of hits in the coincidence window set. The fast processing guarantees that the event detected in FT_K_ time is sent completely including the configured number of pre-event samples.
7. Depending on the configuration applied (searching mode), only Type A, or also Type B defined signals, are considered. If the number of hits and the time between them satisfies the configuration, Event Accept is generated and sent to the DAQ Modules. The Control Module sends to the PC farm data about the configuration set and the sensors involved in the Event Accept generated.
8. Upon arrival of the Event Accept in the DAQ Modules, the end of the buffer is calculated with the Event Accept data sent and the configured buffer size and pre-events. Once the buffer is filled, buffer data is readout and formatted, and sent to the PC farm.

As seen above, the event detection system processors introduce latency that does not affect its functionality due to the large buffer provided and the possibility to send a configurable number of pre- and post-event data.

## 5. System Performance

### 5.1. Data Acquistion

The intensive calibration campaigns, especially with ^83m^Kr sources and their event topology associated, have prioritized the S_2_ signal detection over S_1_ using the Single Searching Mode. This decision was taken based on sheer simplicity as S_2_ signals are noticeably more prominent than S_1_ signals further requiring a less precise configuration. This is especially the case with ^83m^Kr sources as their weak S_1_ signal is hard to distinguish from a dark count-induced response at a given PMT, as can be seen on Figure 6. This, of course, is not the case of the high energy sources where S_1_ triggering would not be as challenging. However, for offline processing simplicity, because the signal that causes the Event Accept is always in the same position in the acquisition window, it is desirable to configure both processors for S_2_ signal detection. Despite this, all the described features, among them the Double Searching Mode, have been successfully tested and validated.

An advantage of the event detection system is the possibility to simultaneously perform very long calibration runs with low energy sources (tens of keV) combined with searches for high energy physics (few MeV) events. The simultaneous data-taking scheme is of vital importance for the experiment and one of the highlights of the acquisition system as it permits to have deep knowledge at all times of the electron lifetime and the geometrical corrections maps, necessary ingredients to correct high energy events and achieve the objective energy resolution as described in [5,16]. Moreover, this scheme is not only important to maximize the detector potential but also to monitor the detector status during the data-taking as the event rate of the high energy events, aside from the calibration periods, is considerably small, of the order of few hundreds of mHz. Therefore, any deviation from the standard operation is considerably harder to notice in the high energy events, and if relaying only on it, the reaction time would be much worse. Once this feature was included and tested properly, NEXT-White has carried out several runs during two years, combining calibration with low and high energy, using ^83m^Kr and ^228^Th sources, low background or double beta decay searches. Around 900 million events have been processed along this period of time. In each run, the low-energy events acquired are used to compute the lifetime and geometric correction maps that are used to correct the high-energy events.

In addition to the simultaneous data-taking, another tool that is proving itself to be greatly beneficial for the experiment, is the capability to keep count of rejected Event Accepts that are considered lost events by the DAQ (see Section 4.4.1). This can be used as an estimate of the dead time of the detector on a run-by-run basis, allowing to correct each run individually by the number of known lost events. Proper understanding of the lost events is required in order to compare the measured background with the expected one and thus correctly extract the half-live of the two neutrino double beta decays over the time for a run.

### 5.2. Dead Time

Another innovative feature reported is the association of the type being searched with the use of a double buffer. This feature has an impact in the dead time for the events selected as EVT1 or EVT2 type, as described in Section 4.1. Table 2 shows three runs taken with the same configuration where dead time increases with data rate, which has been fixed by adjusting the quantity of ^83m^Kr in the chamber. Data rate can be lowered by adjusting the event detection parameters though runs are configured with a wide margin in them so that no bias is expected in the data analysis even at the cost of an increase of the dead time. Moreover, the table shows that almost the total data rate is due to the ^83m^Kr source events, with the beta decay events representing less than 0.5% of the total rate.

Data throughput bottleneck can be easily solved adding more Local and Global Data Concentrator PCs (LDCs and GDCs) to the online system. For the time being, due to the current needs of the detector, a maximum throughput of around 120 MB/s has been fixed in the online hardware configuration described in Section 3.2.

Dead Time improves when only one event processor is used. This is mainly because the double processor shares the double buffer and the GbE links to readout the data. Table 3 shows that dead time for a high energy run can be almost negligible if only one processor is used. In the runs shown, for beta decay events, dead time decreases from 0.46% to 0.03%.

### 5.3. Trigger Efficiency

Trigger efficiency has been also studied, for low and high energy searches, specifically for ^83m^Kr and ^228^Th sources. In both cases, independent events have been obtained from coincidences with other events. This has been done by looking at events that have an additional S_2_ candidate aside from the S_2_ which caused the Event Accept. The additional S_2_ signal has been characterized and only signals which are fully compatible with the event under study have been kept, filtering out any incompatible S_2_. In particular, events have been chosen based on their well-known energy and their position within the buffer window, leaving enough margin from the event accept position (at the middle of the buffer) to avoid interferences. These events are completely uncorrelated with other events in the buffer window and, therefore, are an unbiased sample which can be used to evaluate the event detection efficiency. This strategy has been used for low and high energy events. Selected data have been processed by a Python model of the event detection system. For each type of event under study, individual event search and possible coincidences are set with the same conditions of the runs under study. Event Processor has shown an efficiency of 100%, but as it is shown in Table 4, the Coincidence Event Processor reduces the event detection efficiency for both type of events to a value between 97–98%. Table 4 also shows that there is a margin of improvement to the 98–99% widening the coincidence window to its maximum possible value. In any case, the number of events lost due to event detection efficiency is below the one shown due to dead time.

The efficiency reported integrates all selected events for the study without taking any additional considerations. Full geometrical and energetic characterization will be performed in the future.

## 6. Conclusions

This paper has presented the NEXT-White Event Detection System, adapted from the latter prototype NEXT-DEMO, fully commissioned and deployed in NEXT-White, and ready to be dimensioned (thanks to its modularity) and installed in NEXT-100. The system has evolved during the four years of operation of the detector, being an important tool to prove its event identification capabilities. Its performance, reliability and robustness in the simultaneous calibration and double-beta physics searches have been thoroughly tested.

Among many other, the key feature of NEXT-White, and future NEXT detectors, is the capability of detecting a wide variety of events with the highest efficiency. This fact helps to avoid an excess of data throughput and reduces the amount of offline data to be analyzed. The system described is able to search for events in a very wide dynamic range, triggering in the S_1_ or S_2_ signals of different types of sources, from Kr events that are very low in amplitude and short, to other events with very high amplitude, like S_2_ alpha events, or very long like muons, thanks to the high versatility and configurability of the processors implemented.

Moreover, the number of lost events shows a remarkable improvement when only one event processor is used, as stated in Section 5.2. Anyway, in the case the double processor is used, the number of lost events is low for one type of event, considered of interest. In this sense, the modular hardware used that allows to expand easily the data throughput, as well as the programmability of the implemented system, have both demonstrated its adaptability to the new detector demands.

## 7. Future Work

NEXT final detector is more demanding in terms of readout channels and chamber drift time, which implies more DAQ buffering and processing capacity. The event detection system will need to cope with the processing of more channels, up to 60 PMT signal sensors compared to the 12 of NEXT-White. From this point of view, the advantage of the current modular DAQ and event detection system is that it has been dimensioned from the beginning to cope with the number of sensors that will allocate NEXT-100. Due to the modularity of the RD51 electronics and DATE, DAQ hardware and online system can be easily scaled.

Next step in the NEXT collaboration will be to extrapolate the technology to a 1-ton scale detector. This will imply a deep revision of the detector that will affect completely the DAQ and the event detection system like the replacement of the PMT sensors of the Energy Plane by SiPM sensors. This will imply the study of new detection techniques since the sensors show a completely different behavior. Studies of new detections algorithms based on the concepts learned in the previous detectors must be carried on.

Another line of improvement is the reduction of the dead time in double searching mode (see Section 4.4.1 and Section 5.2). In addition to scaling the online system, a double buffer per type of event could be foreseen. Both could reduce dead time drastically for every type of event. In the same direction, to provide 10 Gb Ethernet connection would speed up data download, reducing dead time as well. DAQ modules have foreseen this type of connection, but the current online system, DATE, does not support any more UDP transactions. For this reason, the use of other online systems, like RCDAQ (PHENIX experiments), is under study. In the short term, next version of DAQ will provide a configurable size of buffer per event type. This will reduce the data throughput for low energy events in the simultaneous calibration and double-beta physics searches.

## Figures and Tables

**Table 1:** Event detection parameters applied to detect some of the calibration sources, muons and beta decay physics during RUN V. Same configuration applied in the event processor for all the PMT sensors. Circular Buffer size of 1600 µs and pre-trigger of 800 µs.

Event Type	Event Processor Parameters						Coincidence Event Processor Parameters	
	Energy		Amplitude		Time		Time Coincidence Window	Coincident Events
	Accumulation of ADC Counts		Relative to the Baseline		µs		µs	Number of PMTs
	Min	Max	Min	Max	Min	Max		
ββ	100,000	16,777,215 ^1^	10	-	2	600	1.2	2
^83m^Kr	5000	50,000	10	1000	2	40	1.2	2
^232^Th	220,000	16,777,215	10	2000	10	250	0.5	2
Muons	100,000	16,777,215	10	-	2	600	1.2	2

^1^ Maximum possible value (24 bits parameter).

**Table 2:** Three run statistics with general configuration: Single Searching Mode, two type of S_2_ events (^83m^Kr and beta decay events), 24 h, buffer priority for beta decay events, at least 2 PMT hits in a Coincidence Window of 1.2 µs, data compression applied.

	Total Event Rate	Data Rate	EVT Rate	Dead Time Rate	
			EVT2	EVT1	EVT2
7520	20.78 Hz	31.78 MB/s	1.95 Hz	9.4%	3.8%
7512	27.27 Hz	41.66 MB/s	1.01 Hz	16.4%	5.3%
7502	76.53 Hz	77.28 MB/s	0.84 Hz	39.2%	15.15%

**Table 3:** Two run statistics with general configuration: Single Searching Mode, 24 h, buffer priority for beta decay events, at least 2 PMT hits in a Coincidence Window of 1.2 µs, no data compression applied. RUN 8087 is set for two type of S_2_ events (^83m^Kr and beta decay events) and RUN 8091 is set for one type of S_2_ events (beta decay events).

RUN	Total Event Rate	Data Rate	EVT Rate	Dead Time Rate	
			EVT2	EVT1	EVT2
8087	8.56 Hz	26.25 MB/s	0.089 Hz	1.06%	0.46%
8091	0.097 Hz	0.31 MB/s	0.097 Hz	-	0.03%

**Table 4:** Coincidence Event Processor efficiency results. Configuration under study: Two minimum PMT hits in a Coincidence Window (CW) of 1.2 µs (run parameter) and 1.6 µs (maximum possible value).

RUN	Event Type Searched	Number of Events	Event Detection Efficiency	
			CW = 1.2 µs	CW = 1.6 µs
7492	^83m^Kr	41,101	97.33%	98.25%
7738	^228^Th	3511	98.15%	99.09%

## Data Availability

Data collected through research presented in the paper are available on request from the corresponding authors.
